# Pneumonia in low and middle income countries: progress and challenges

**DOI:** 10.1136/thoraxjnl-2013-204247

**Published:** 2013-08-16

**Authors:** H J Zar, S A Madhi, S J Aston, S B Gordon

**Affiliations:** 1 Department of Paediatrics and Child Health, Red Cross War Memorial Childrens Hospital, University of Cape Town, Cape Town, South Africa; 2 National Institute for Communicable Diseases, Division of National Health Laboratory Service, Johannesburg, South Africa; 3 Medical Research Council: Respiratory and Meningeal Pathogens Research Unit & Department of Science/National Research Foundation: Vaccine Preventable Diseases, Faculty of Health Science, University of the Witwatersrand, Johannesburg, South Africa; 4 Malawi Liverpool Wellcome Trust Clinical Research Programme, Blantyre, Malawi; 5 Liverpool School of Tropical Medicine, Liverpool, UK

**Keywords:** Pneumonia

## Abstract

Pneumonia remains the leading cause of childhood mortality and the most common reason for adult hospitalisation in low and middle income countries, despite advances in preventative and management strategies. In the last decade, pneumonia mortality in children has fallen to approximately 1.3 million cases in 2011, with most deaths occurring in low income countries. Important recent advances include more widespread implementation of protein-polysaccharide conjugate vaccines against *Haemophilus influenzae type B* and *Streptococcus pneumoniae*, implementation of case-management algorithms and better prevention and treatment of HIV. Determining the aetiology of pneumonia is challenging in the absence of reliable diagnostic tests. High uptake of new bacterial conjugate vaccines may impact on pneumonia burden, aetiology and empiric therapy but implementation in immunisation programmes in many low and middle income countries remains an obstacle. Widespread implementation of currently effective preventative and management strategies for pneumonia remains challenging in many low and middle income countries.

## Introduction

Pneumonia is a major cause of morbidity and mortality in children and adults in low and middle income countries (LAMICs). In the last decade there have been several advances and new interventions, resulting in a substantial reduction in pneumonia incidence and improved outcomes. These include more widespread use of case management strategies, development and implementation of polysaccharide-protein conjugate vaccines, better prevention of HIV transmission and uptake of effective antiretroviral therapy (ART) of HIV-infected adults and children. As a result there has been a considerable reduction in pneumonia mortality in children under 5 years of age, from 1.7 million cases globally in 2000 to 1.3 million cases in 2011.^1^ Nevertheless, pneumonia remains the major cause of death in children worldwide beyond the neonatal period; this is especially concerning as most pneumonia deaths should be preventable.^2^ Further, pneumonia remains the most common reason for adult hospitalisation in sub-Saharan Africa, with an estimated 4 million episodes and 200 000 deaths each year.^3^

## Childhood pneumonia

### Epidemiology and aetiology

In 2011, there were an estimated 120 million episodes of childhood pneumonia globally, of which 14 million progressed to severe disease, with 1.3 million deaths.^1^ Most deaths (81%) occurred in children under 2 years of age.^1^ The incidence and severity of childhood pneumonia was highest in Africa and southeast Asia, which accounted for 30% and 39% respectively of the global burden of severe cases.^1^ In these two regions, 15 countries accounted for two-thirds of all childhood pneumonia episodes and severe cases.^1^ Estimates of hospitalisation for acute lower respiratory tract infection (ALRI) in children in 2010 provide an indication of the large burden of disease with almost 12 million hospitalisations for severe disease and 3 million for very severe infection.^4^ Even more concerning is the estimate that more than 80% of deaths occurred outside a hospital; 99% of deaths occurred in LAMICs.

Risk factors for pneumonia and for severe disease include poor nutrition including micronutrient deficiency, lack of breastfeeding, exposure to indoor air pollution or passive smoke exposure, HIV infection, premature birth, overcrowding and poor living circumstances.^5–8^ HIV-infection is a particularly important risk factor in children in sub-Saharan Africa where the burden of paediatric HIV disease is concentrated.^7^ Children with HIV infection develop more severe pneumonia, have a higher mortality and have an increased risk of pneumonia from opportunistic organisms such as *Pneumocystis jirovecii* and cytomegalovirus.^7^

Identification of the aetiology of pneumonia is challenging as few children develop bacteraemic illness and high prevalence of nasopharyngeal colonisation by potentially pathogenic bacteria, limits the use of respiratory samples for diagnosing bacterial pneumonia. Nevertheless data from vaccine-probe studies indicate that the predominant aetiologic agent is *Streptococcus pneumoniae*, which is estimated to cause 18% of severe cases and 33% of deaths.^1^ ^9^ Other important vaccine preventable pathogens include *Haemophilus influenzae* type b (Hib), estimated to account for 4% of severe episodes and 16% of deaths and influenza virus which is associated with approximately 7% of severe episodes and 11% of deaths.^1^ With improved vaccine uptake, the importance of the vaccine-targeted pathogens is anticipated to diminish,^9^ while a greater proportion of cases may occur due to *Staphylococcus aureus, Klebsiella pneumoniae* and *Mycobacterium tuberculosis* in tuberculosis (TB) endemic areas.

Other respiratory viruses, other than influenza virus, also contribute considerably to the burden of childhood pneumonia. Globally, an estimated 100 million cases of viral-associated pneumonia occur annually in children; respiratory syncytial virus (RSV), rhinovirus, human metapneumovirus, human bocavirus, and parainfluenza viruses are the most common viral pathogens identified in affluent and in LAMICs.^9^ ^10^ In 2005, RSV was estimated to cause approximately 34 million episodes of ALRI in children under 5 years or 22% of all ALRI; 10% of episodes resulted in severe illness and hospitalisation and 99% of deaths occurred in LAMICs.^11^ With improved immunisation against the main bacterial pathogens, respiratory viruses may become more prominent as aetiologic agents of pneumonia. Moreover, current evidence suggests severe pneumonia results from infection with multiple pathogens such as bacterial-viral, dual viral or mycobacterial-bacterial infections.^9^ ^10^ Up to a third of children with pneumonia may have viral-bacterial co-infections.^10^

### Treatment

The cornerstone of effective treatment for childhood pneumonia remains appropriate antibiotics and supportive care including oxygen.^2^ ^12^ Use of oxygen systems in children with hypoxic pneumonia can reduce mortality by approximately 20%.^13^ Use of the pneumonia case management strategy included in the World Health Organisation Integrated Management of Childhood Illness (IMCI) programme has consistently been reported to reduce childhood mortality by approximately 20%, with even higher reductions in pneumonia specific mortality.^14^ Community based case management of childhood pneumonia may reduce pneumonia mortality by 70%.^15^ Accumulating evidence suggests that community based use of oral antibiotics for severe pneumonia may be a feasible and effective strategy for reducing mortality.^12^ ^16^ ^17^ Furthermore, short course antibiotic therapy (3 rather than 5 days) has been reported to be effective for pneumonia in immunocompetent children.^18^

The reduction of Hib and pneumococcal associated pneumonia through use of conjugate (HibCV and PCV) immunisation, underscores the need to reconsider the empiric treatment of pneumonia in settings where there are strong national immunisation programmes. With high coverage of HibCV, Hib is unlikely to contribute to a sizeable portion of pneumonia. The relative ongoing role of pneumococcus as an aetiologic agent for pneumonia, however, remains to be fully elucidated and may vary depending on the prevalence of disease causing non-vaccines serotypes. The impact of PCV and HibCV on the aetiology of childhood pneumonia in LAMICs is currently being evaluated in a large multicentre study, the Pneumonia Etiology Research for Child Health (PERCH) study.^6^

### Prevention in the era of conjugate vaccines

Improved access to health care, better nutrition, promotion of breast feeding, improved living conditions and reduced exposure to indoor pollutants may contribute to the reduction in incidence of pneumonia and decline in case fatality rates.^2^ ^6^ ^8^ In areas of south east Asia and Latin America, high exposure to biomass fuel remains an important factor impacting on the incidence and severity of childhood pneumonia, while in many LAMICs passive exposure to cigarette smoke is highly prevalent.^8^ Improved home ventilation and reduction in exposure to indoor air pollution and cigarette smoke are important strategies to reduce the severity and incidence of childhood pneumonia. For HIV-infected children, use of ART early in the course of HIV infection and of cotrimoxazole prophylaxis can substantially reduce the burden of pneumonia and of severe disease.^7^

Prevention of pneumonia has also been expedited by the introduction of HibCV and PCV.^2^ Combined data from six studies of the effectiveness of HibCV in LAMICs indicates a reduction of 18% in radiological pneumonia, of 6% in severe pneumonia and of 7% in pneumonia-associated mortality.^2^ Progress toward the inclusion of HibCV into public immunisation programmes lagged behind in low income countries, with HibCV introduced in <25% of low-income countries by 2006, almost 20 years since its licensure in developed countries.^19^ More recently, through support from Global Alliance for Vaccines and Immunisation (GAVI), approximately 90% of low-income countries had introduced HibCV in 2011.^19^

A conservative estimate of the role of pneumococcus in childhood pneumonia has also been possible through vaccine-probe studies. Overall, data from six studies in LAMICs estimated a reduction of 29% in radiologically confirmed pneumonia, of 11% in severe pneumonia and of 18% in pneumonia-specific mortality.^2^ Notably, the prevention of non-bacteraemic pneumococcal pneumonia was 20-fold greater compared to that of vaccine-serotype bacteraemic pneumonia.^20^ ^21^ The reduction in radiologic-confirmed pneumonia from PCV is mainly due to reductions in vaccine-serotype disease and occasionally some cross-reactive serotypes. Consequently, the use of PCV as a vaccine-probe may underestimate the overall contribution of pneumococcus as an aetiologic agent for radiologic-confirmed pneumonia.

Data from the PCV9 study among HIV-uninfected South African children reported vaccine efficacy against vaccine-serotype invasive disease (IPD) of 83% and 35% against all serotype IPD, with a 20% reduction in radiologic-confirmed pneumonia.^21^ Based on the assumption that the serotypes causing pneumonia are similar to those causing IPD, it is likely that PCV9 only prevented one-third of non-bacteraemic pneumococcal pneumonia; therefore a significant proportion (possible up to two-thirds) of the residual burden of radiologic-confirmed pneumonia among PCV-vaccinated children may still be due to pneumococcus either due to incomplete protection against vaccine-serotype pneumonia, or due to lack of efficacy against non-vaccine serotypes. Despite these draw-backs, PCV has resulted in a dramatic decline in all-cause pneumonia hospitalisation in several countries, especially in children less than 2 years of age. This included a 56% reduction following 7-valent PCV introduction in a middle-income country such as Uruguay,^22^ as well as a sustained reduction (43% decrease) in children under 2 years in USA,^23^ 10-years following PCV introduction. The reduction of pneumonia in USA among children 2–4 years of age has, however, been more modest (12%).^23^ Furthermore, widespread PCV immunisation of infants has also led to a dramatic decline in the rates of hospitalisation for pneumonia in adults, especially the elderly.^23^ ^24^ PCV has also been associated with reduction in hospitalisation for viral-associated pneumonia. A post hoc analysis of the South African PCV9 study found a 32% lower hospitalisation rate for pneumonia episodes associated with respiratory viruses in vaccinated children.^25^ Effectiveness studies in countries with widespread PCV immunisation have corroborated these findings for example, a 41–50% reduction in influenza virus attributable pneumonia hospitalisation for every 10% increment in childhood PCV immunisation in the USA.^24^ A case-control study in Spain reported a 48% reduction for influenza hospitalisation associated with the H1N1 pandemic in PCV fully-vaccinated children during 2009–2010, however, a similar protective effect against seasonal-influenza virus hospitalisation in 2010–2011 was not found.^26^ Furthermore, PCV vaccination of infants has also provided insight into the role of pneumococcus in precipitating hospitalisation for acute severe pneumonia in children with underlying TB in TB endemic settings. Another post hoc analysis of the South African PCV9 trial indicated that 43% of hospitalisation in children with culture-confirmed TB was due to a pneumococcal co-infection.^27^

Progress has also been made in developing vaccines which are more immunogenic and efficacious against influenza virus. These include the live attenuated influenza vaccine (LAIV) and MF-59 adjuvanted inactivated influenza vaccines, both of which have a higher relative efficacy compared to non-adjuvanted inactivated influenza vaccine (IIV) against influenza confirmed illness. Among children age 12–24 months old, the relative efficacy of LAIV was 55% compared to IIV,^28^ and in children 6–72 months of age adjuvanted IIV had a 75% higher relative efficacy in preventing influenza confirmed illness compared to IIV.^29^

## Adult pneumonia

### Epidemiology

Pneumonia is the most common cause of infectious disease related death among adults worldwide and is associated with age, HIV infection and smoking. In LAMICs, where life expectancy is much lower than in developed economies, the dominant risk factors are HIV and smoke exposure.

The association of pneumonia and HIV infection is CD4 dependent with exponential increases in incidence with decreasing CD4.^30^ ART roll-out in many resource poor settings has reduced the incidence of pneumonia among HIV infected adults but anti-pneumococcal immunity is not fully restored by ART.^31^ ^32^ It is therefore unsurprising that in HIV endemic areas, pneumonia remains the most common cause of inpatient admission as many people are only diagnosed as HIV infected at presentation with pneumonia and progression to AIDS remains common.^33^ Pneumonia outcomes among HIV infected people have been reported as similar to HIV uninfected patients given optimal therapy but patients with HIV often present with complex co-infections and co-morbidity.^34^

Smoke exposure in developed countries is predominantly tobacco related but in LAMICs there is an equal burden of disease attributed to household air pollution. The association of upper and lower respiratory tract infection with biomass fuel exposure has been known for some time^35^ but there are no data to confirm that an intervention to reduce smoke exposure can reduce adult pneumonia. Intense efforts to reduce biomass fuel consumption and household air pollution have been driven by political will to reduce environmental destruction, ameliorate climate change and empower women—the evidence to support a health effect, and the data with which to choose appropriate technology are urgently needed.

### Aetiology of adult pneumonia in resource poor settings

Whilst *S pneumoniae* is a universally common cause of adult pneumonia globally, there are marked regional differences in the frequencies of other pathogens that are important to consider when selecting empirical antimicrobial treatment or determining the need for additional microbiological investigations. In many low-resource settings, there is a particularly high incidence of pulmonary TB. In Kenya, for example, *M tuberculosis* was the second most commonly identified pathogen in a large prospective cohort study of adults with pneumonia and notably symptoms of 14 days or less.^36^ In China and several other Asian countries, high rates of pneumonia attributable to atypical bacterial pathogens, particularly *Mycoplasma pneumoniae*, are commonly reported and macrolide resistance is a growing problem.^37^ ^38^ *K pneumoniae* has emerged as a frequent cause of severe pneumonia in both Asia and South Africa and substantial rates of multi-drug resistance have been observed.^39^ ^40^ *Burkholderia pseudomallei*, the causative agent of melioidosis, is endemic throughout much of South-East Asia and frequently presents as an acute severe pneumonic illness. In some rural areas, it is the most commonly identified cause of severe pneumonia.^41^ The emergence of several novel respiratory viral infections from LAMICs in recent years serves to highlight the importance of continued vigilance to novel pathogens in patients presenting with acute pneumonia.

### Pathogen specific diagnostic tests in adult pneumonia

In developed settings, treatment algorithms based on aetiology have failed to show a therapeutic benefit and this has driven clinical apathy towards precise microbiological diagnosis.^42^ In settings where HIV infection is found in most adults presenting with pneumonia, however, it is probable that pathogen directed care, at least to distinguish bacterial infection from TB or *P jirovecii* pneumonia, will provide substantial benefit. In the setting of severe disease, accurate positive or negative results would be extremely useful in ruling treatment options in or out. This need for diagnostic precision and regionally relevant management strategy is the currently the subject of focused cohort studies.

The role of respiratory viruses in both HIV infected and uninfected adults in resource poor settings is unknown and is the subject of intense current research effort. Surveillance studies, led by Centers for Disease Control and Prevention (CDC), USA and clinical outcome prospective cohorts are in progress. The interaction of influenza and bacterial infection discussed above with regard to child vaccination is likely to also pertain in adults.^43^ It is unlikely, however, that current viral or bacterial diagnostic tests would alter therapy even in resource poor settings.

## Management, treatment and antibiotic regimens in adult pneumonia

### Severity scores

Appropriate initial management of pneumonia relies on an accurate assessment of disease severity. To support clinical assessment, which is poorly predictive of disease outcome, several severity-scoring indices have been developed. These tools facilitate the prompt identification of individuals with severe disease, permitting the rapid institution of intensive care interventions. Equally, they help treating physicians to confidently identify individuals with low-severity disease who may be safely managed at home. Care provided in accordance with treatment guidelines based on an assessment of disease severity using a severity-scoring index is associated with improved outcome.^44^ Nonetheless, many severity assessment tools may actually underestimate disease severity in young people.^45^ This is of particular relevance in LAMICs where a greater burden of pneumonia is seen in younger adults.^36^ Similarly, differences in population comorbidity burden and aetiological spectrum of pneumonia may reduce the prognostic performance of most pneumonia severity assessment tools that were almost exclusively developed and validated in well-resourced settings. There is an urgent need for the development of validated severity assessment tools in LAMICs that can support clinical management decisions in these settings.

## Treatment

In the absence of data from high quality randomised controlled trials, the optimal antimicrobial treatment for moderate or high severity community-acquired pneumonia remains uncertain. Observational studies support the use of combination antimicrobial therapy with a β-lactam and macrolide.^46^ The beneficial effect of inclusion of macrolide antibiotics are not limited to pneumonia caused by atypical bacteria, but are also evident in severe pneumococcal pneumonia and disease due to macrolide-resistant pathogens,^47^ suggesting that non-antimicrobial effects of macrolides may be relevant. The growing interest in the putative immunomodulatory properties of macrolide antibiotics in the treatment of pneumonia is tempered by concerns over their cardiovascular safety profile.^48^ The role of adjunctive immunomodulatory therapies in the treatment of pneumonia more generally remains undefined. Prospective studies of corticosteroids have yielded variable and inconclusive results.^49^ The need for improved therapies remains pressing given the lack of substantial advancement in outcomes of severe pneumonia in the last decades.

Notwithstanding this, recent studies have demonstrated that there are still substantial gains to be made in improved treatment outcomes in pneumonia by the implementation of simple best practice, particularly rapid measurement and correction of hypoxia, and prompt provision of appropriate antibiotics and intravenous fluids.^50^ This care-bundle approach has been successfully used to improve outcomes in severe sepsis and the parallels to the treatment of pneumonia are clear. In resource poor settings, however, basic resuscitation is often difficult and in particular, oxygen provision is minimal.^51^ Despite these challenges, this approach presents an important therapeutic opportunity that is the focus of current research.

Indiscriminate use of broad spectrum antibiotics has been appreciated as a major cause of developed country hospital morbidity due to *Clostridium difficile*.^52^ This burden of iatrogenic disease has largely been avoided in low resource settings owing to the more parsimonious use of antibiotics in general and broad spectrum antibiotics in particular. The future spectre of increased antibiotic resistance in settings with limited ability to purchase sophisticated new drugs is a current worry, however, as is the relative paucity of antibiotics in development.

In well-resourced settings, there is a growing body of evidence demonstrating a continuing excess mortality that persists for many months after surviving an episode of pneumonia.^53^ Increased rates of cardiovascular events have been implicated as the predominant explanation with coronary artery plaque destabilisation related to the intense systemic response during pneumonia representing a plausible biological mechanism.^54^ Strategies to mitigate this risk with the use of aspirin and HMG CoA reductase inhibitors are the focus of current interest. As the burden of coronary-artery disease increases in LAMICs, these long-term health implications of pneumonia may become more apparent.

### Prevention of pneumonia in adults

Globally, vaccination of adults is poor. This is particularly the case in resource-poor settings but, fortunately, there is a substantial impact to be gained in prevention of adult disease from the vaccination of children. This has been dramatically demonstrated in the USA with PCV but also shown to be region and vaccine-specific with the UK experience showing less herd immunity.^55^ ^56^ Vaccination of HIV infected adults to prevent invasive pneumococcal disease was a success using conjugate vaccine but failed using polysaccharide vaccine.^57^ ^58^ This paradoxical result where a T cell dependent vaccine was successful even in severely immunocompromised adults has not been followed by widespread adoption of vaccination of HIV infected adults. Current research is focused on the timing of post-ART vaccination and on the herd protection effects achievable with polyvalent conjugate vaccination of children in communities with high HIV seroprevalence. Pneumococcal vaccination in the context of influenza pandemic will have a role but this has not yet been defined, nor has the need for influenza vaccination in HIV infected people.

## Conclusions

Pneumonia continues to present a major burden of morbidity and mortality in resource poor regions despite advances in preventative and management strategies. There is an urgent need for improved diagnostics to reliably identify aetiological agents. More widespread implementation and scale up of immunisation with improved conjugate vaccines against childhood pneumonia in LAMICs could substantially reduce childhood mortality and the burden of pneumonia. Implementation of current effective preventative and management strategies remains a challenge in many LAMICs although the available interventions can substantially reduce pneumonia burden, severity and outcome.
